# Case on a Wound of the Head, Attended with Singular Phenomena

**Published:** 1818-02

**Authors:** Baron Larrey


*For the London Medical and Physical Journal.*

### *Case on a Wound of the Head, attended with singular Phenomena;* by BARON LARREY.

EDWARD DE RAMPAN, 26 years old, received, while fencing, a stroke of a foil, (the point of which broke on his mask,) near the side of the nose, in an oblique direction from below upwards, and a little from without inwards. The instrument penetrated to the depth of about three inches and a half across the left nasal foss, traversed the cribriform lamina of the ethnoid, near the insertion of the falx cerebri, and appears to have penetrated in a vertical direction, and a little obliquely from before backwards, to the depth of five or six lines in the posterior internal part of the left anterior lobe of the brain, so as to have approached the anterior part of the *mesolobe.*

The patient had a very considerable hæmorrhage at the very moment of the wound, and a great quantity of splinters came out of his nose and mouth. All the organs of the senses were paralysed at the very instant of the stroke; but they resumed their functions by degrees, and there now remain only the following alterations.

The sight of the left eye was entirely lost for a month; but is now restored, only the patient sees objects double.

The smell was quite gone, but is re-established, and he can distinguish odorous alcoholic liquors from inodorous liquids.

The taste was also destroyed; it returned by degrees on the right side of the tongue, so that the right half of that organ perceives tastes very well, while the left half is deprived of that faculty. The whole of this organ is drawn towards the right in opposition to the hemiplegia, which exists on the right side, the mouth declining on the left side.

The hearing, lost at first in the ear on the wounded side, is restored, but a humming remains.—The voice, which was also lost, returned likewise; and there only remains a slight stammering.

The power of the organs of generation is entirely preserved.

A hemiplegia came on the whole right side, but there remains now only a paralysis of the thoracic and abdominal muscles of the same side, with respect to the faculty of locomotion only, its sensibility remaining uninjured.

The recollection of names is lost, or the patient has very great difficulty in recalling them; while the remembrance of images, and of whatever is susceptible of demonstration is perfectly entire.

The mental aberration, which existed at first in the organs of the intellect, has now ceased; but, all that relates to his private feelings, his military prospects, &c. throws him into a state of alienation and profound melancholy; but conversations relative to his family, his relations, and friends, make him recover his faculties.

The patient recollected perfectly well the person, the face, and the features, of M. Larrey; he would have known him again directly; he had him always before his eyes, (the patient's own expression); and yet he could not remember his name, but called him *M. Chose.*

*Paris; Jan.* 1818.

